# Musculoskeletal imaging in disseminated fusariosis: A case report with diagnostic imaging challenges

**DOI:** 10.1016/j.radcr.2026.10.028

**Published:** 2026-11-06

**Authors:** Marina Urbano-Rodríguez, Marta Porta Vilaró, Carlos Benavente Fernández, Gerard Frigola Quintana, Javier Gil-Lianes, Andrés Perissinotti, Ana Isabel García-Díez

**Affiliations:** a Department of Radiology, Hospital Clínic de Barcelona, Spain; b Department of Pathology, Hospital Clínic de Barcelona, Spain; c Department of Dermatology, Hospital Clínic de Barcelona, Spain; d Department of Nuclear Medicine, Hospital Clínic de Barcelona, Spain; e Biomedical Research Networking Center of Bioengineering, Biomaterials and Nanomedicine (CIBER-BBN), Instituto de Salud Carlos III (ICSIII), Barcelona, Spain; f Institut d’Investigacions Biomèdiques August Pi i Sunyer (IDIBAPS), Barcelona, Spain

**Keywords:** *Fusarium solani*, Invasive fusariosis, Hematologic malignancy, Musculoskeletal fungal infection, Multimodality imaging, Immune reconstitution inflammatory syndrome

## Abstract

Disseminated fusariosis is a life-threatening opportunistic fungal infection that primarily affects immunocompromised patients with hematologic malignancies. Its imaging manifestations are heterogeneous and may evolve during immune recovery, complicating the distinction between active infection and inflammatory response. We report the case of a 31-year-old man with acute myeloid leukemia who developed disseminated *Fusarium solani* infection involving the skin, soft tissues, paranasal sinuses and bones during induction chemotherapy. Contrast-enhanced computed tomography, ultrasound, magnetic resonance imaging and ^18^F-fluorodeoxyglucose positron emission tomography/computed tomography (^18^F-FDG PET/CT), demonstrated lesion evolution after neutrophil recovery, including a characteristic ultrasound target sign and corresponding peripheral enhancement and FDG uptake despite clinical improvement. Histopathology and follow-up biopsies supported immune reconstitution inflammatory syndrome rather than persistent fungal infection. This case highlights the importance of interpreting imaging findings with clinical and pathological data during immune recovery. Recognition of imaging changes associated with immune reconstitution inflammatory syndrome may help avoid misdiagnosing inflammatory lesions as persistent or progressive fungal infection.

## Introduction

Disseminated fusariosis is a rare but life-threatening opportunistic infection, primarily affecting patients with hematologic malignancies undergoing induction chemotherapy or hematopoietic stem cell transplantation [1,2]. Profound neutropenia markedly increases susceptibility to invasive fungal infections, and early diagnosis remains challenging due to nonspecific clinical presentation [3].

Invasive fusariosis is defined by either positive blood cultures or involvement of at least 2 distinct organs [1]. This condition carries a poor prognosis, with reported mortality rates approaching 60%-70%. Clinical presentation most commonly includes febrile neutropenia with cutaneous lesions, and may involve the paranasal sinuses, lungs, and less frequently the central nervous system. Musculoskeletal involvement is uncommon, and its imaging spectrum remains poorly characterized [4,5].

We report a case of disseminated fusariosis in a patient with acute myeloid leukemia, highlighting the evolving multimodality imaging findings during immune reconstitution, including imaging “target sign” on ultrasound, and emphasizing the importance of radiologic-pathologic correlation.

## Case presentation

A 31-year-old man presented to the emergency department with fever (39°C), diffuse erythematous papular skin lesions (Fig. 1A), and severe pain in the left leg. His medical history included a right lower-limb amputation for childhood Ewing sarcoma. He was in complete remission, with no residual immunosuppression related to his previous malignancy, and was receiving induction chemotherapy for acute myeloid leukemia.

**Fig. 1:**
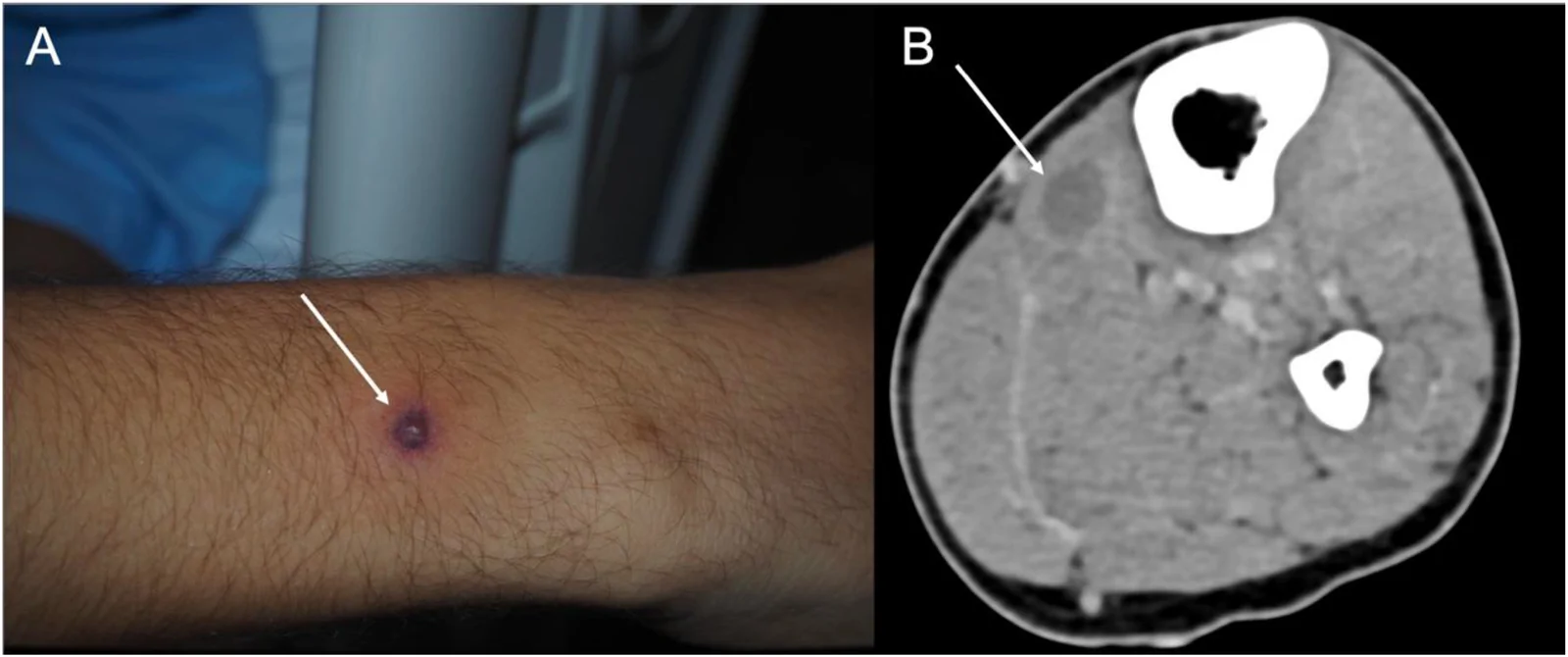
(A) Clinical image of the left arm showing diffuse, painful violaceous papules with surrounding erythema (white arrow). (B) Axial contrast-enhanced CT of the lower left leg demonstrating intramuscular hypodense lesions with peripheral rim enhancement in the soleus muscle (white arrow), consistent with early soft-tissue involvement.

He was hemodynamically unstable (blood pressure 60/40 mmHg, heart rate 130 bpm). Laboratory tests revealed severe neutropenia (absolute neutrophil count 10/µL), anemia (hemoglobin 7.5 g/dL), thrombocytopenia (11 × 10³/µL), and elevated C-reactive protein (31 mg/dL). Febrile neutropenia with septic shock was diagnosed, and supportive therapy included intravenous fluids, granulocyte colony-stimulating factor, blood transfusions, empiric meropenem, and isavuconazole. Chemotherapy was suspended.

Skin biopsy of the papular lesions demonstrated thrombotic vasculopathy, and PAS and Grocott staining revealed numerous intravascular and perivascular fungal hyphae.

Due to persistent left-leg pain and swelling, contrast-enhanced CT showed multiple intramuscular hypodense lesions with peripheral rim enhancement in the gastrocnemius and soleus muscles, consistent with abscesses (Fig. 1B). Ultrasound excluded deep vein thrombosis and revealed hyperechoic, well-defined soft-tissue nodules (Fig. 2A). Ultrasound-guided biopsy yielded 4 muscle specimens (Fig. 2B). Histopathology revealed abundant fungal elements within the endomysial, perimysial and intravascular compartments, with acute-angle branching and positive PAS and Grocott staining, confirming angioinvasive infection (Fig. 2C and D). Microbiological cultures identified *Fusarium solani*.

**Fig. 2:**
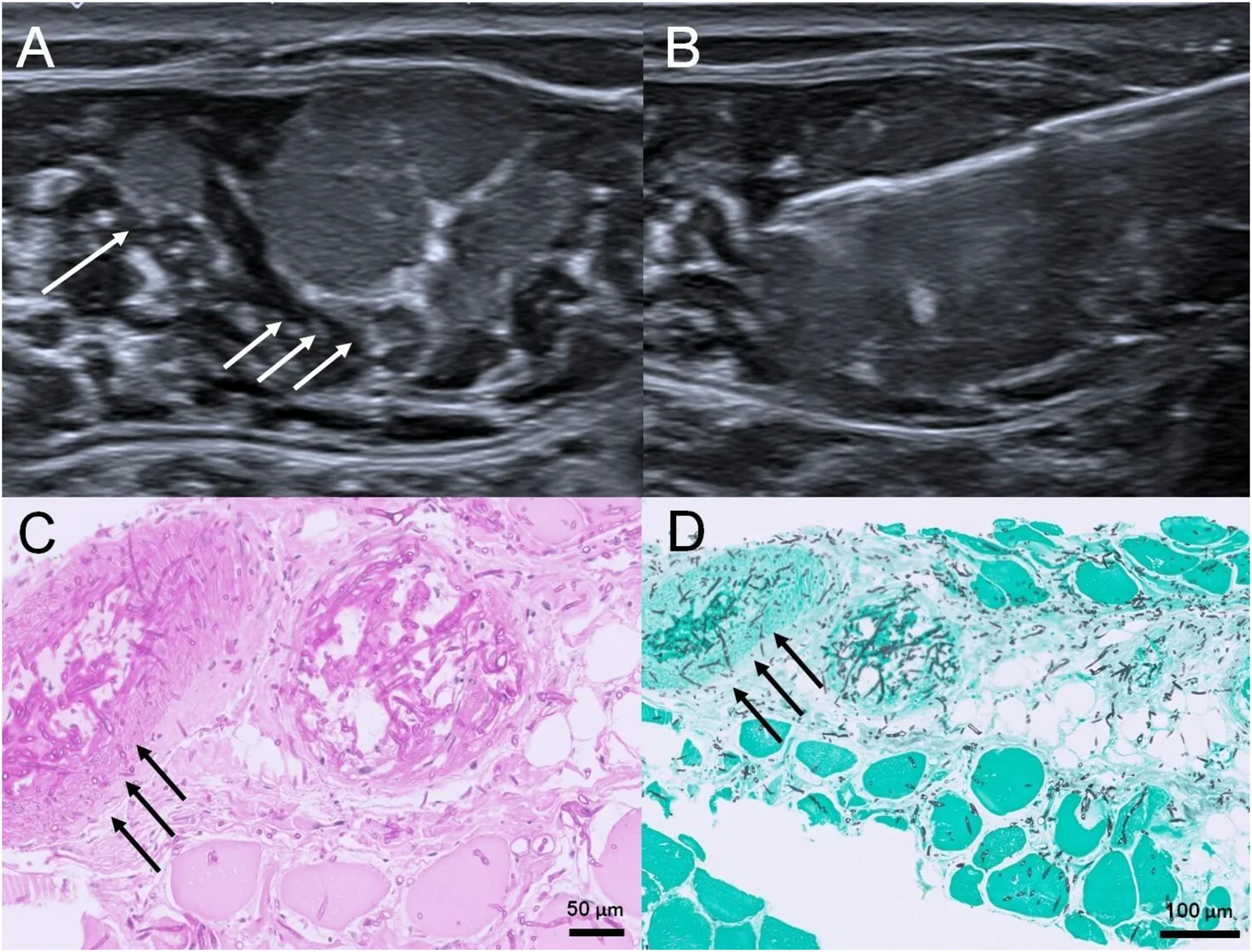
(A) Ultrasound of the left leg showing rounded, well‑circumscribed hyperechoic intramuscular lesions (white arrows). (B) Ultrasound‑guided biopsy of the largest lesion. (C, D) Histopathology (PAS and Grocott methenamine silver stains) demonstrating numerous fungal hyphae with intravascular involvement (black arrows).

To evaluate disease extent, ^18^F-fluorodeoxyglucose positron emission tomography/computed tomography (^18^F-FDG PET/CT) was performed. Multiple hypermetabolic soft-tissue lesions were identified in the right retroauricular region, both forearms, and the left lower extremity. Most lesions demonstrated central hypoactivity, suggestive of abscess formation (Fig. 3A). Pathological uptake was also noted in the right ethmoid air cells with associated soft-tissue thickening (Fig. 3B). These findings were consistent with disseminated fusariosis. No pulmonary or intra-abdominal involvement was detected.

**Fig. 3:**
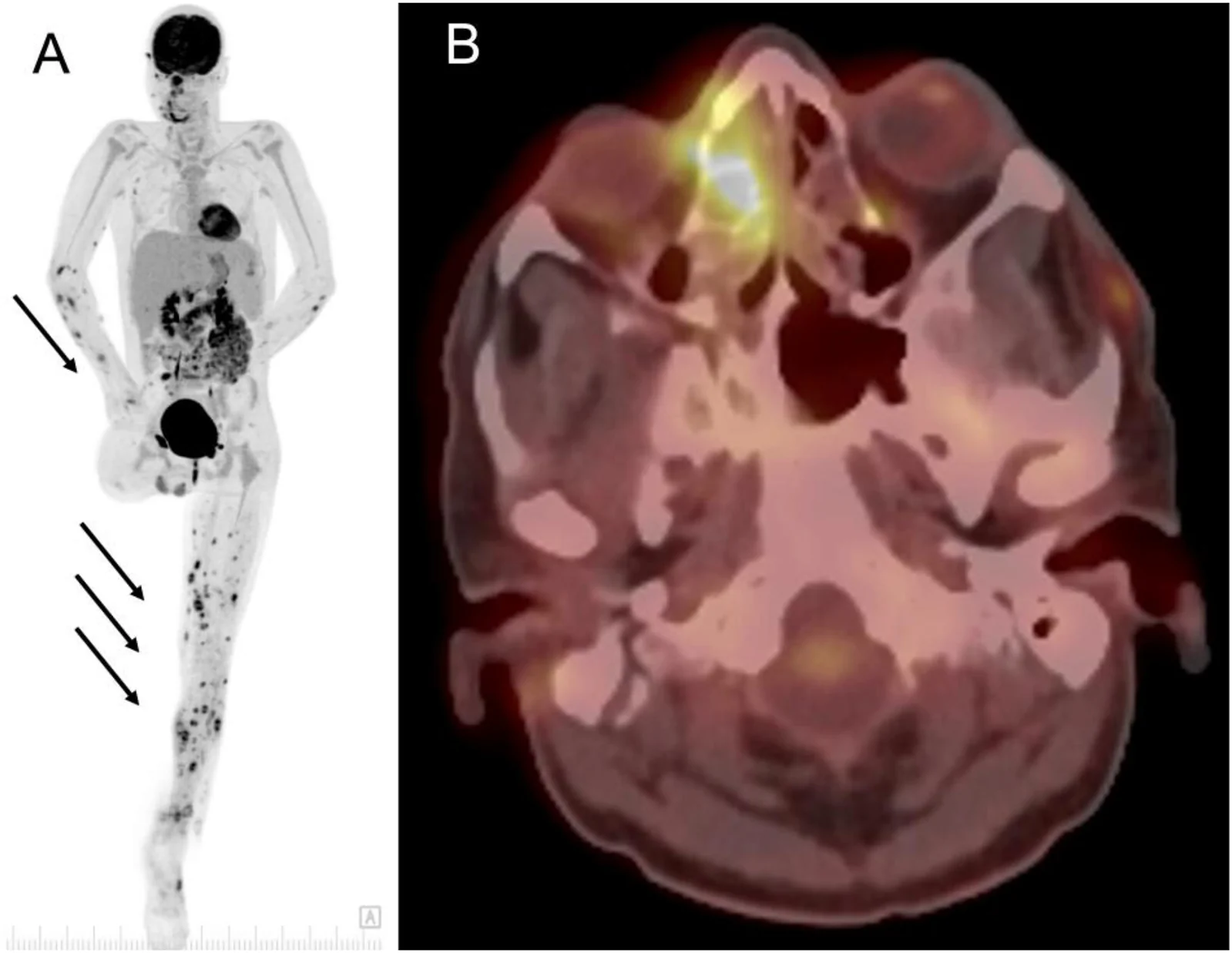
(A) Initial ^18^F-FDG PET/CT showing multiple hypermetabolic soft‑tissue lesions in both forearms and the left lower extremity. (B) Pathological uptake is also noted in the right ethmoid sinus associated with soft tissue thickening, consistent with disseminated fungal infection.

Antifungal therapy was escalated to liposomal amphotericin B, terbinafine, isavuconazole, and fosmanogepix (compassionate use), and the patient underwent endoscopic sinonasal debridement.

Despite clinical improvement and hematologic recovery, follow-up imaging performed 2 weeks after initiation of antifungal therapy, coinciding with neutrophil recovery, demonstrated an increase in the number and size of lesions. On ultrasound, lesions evolved into rounded nodules with a characteristic “target sign,” consisting of a hyperechoic center surrounded by a hypoechoic halo with increased Doppler signal (Fig. 4A). CT showed interval progression of intramuscular lesions with multilobulated morphology and peripheral rim enhancement (Fig. 4B), while ^18^F-FDG PET/CT revealed persistent peripheral hypermetabolism in soft-tissue lesions (Fig. 4D and E).

**Fig. 4:**
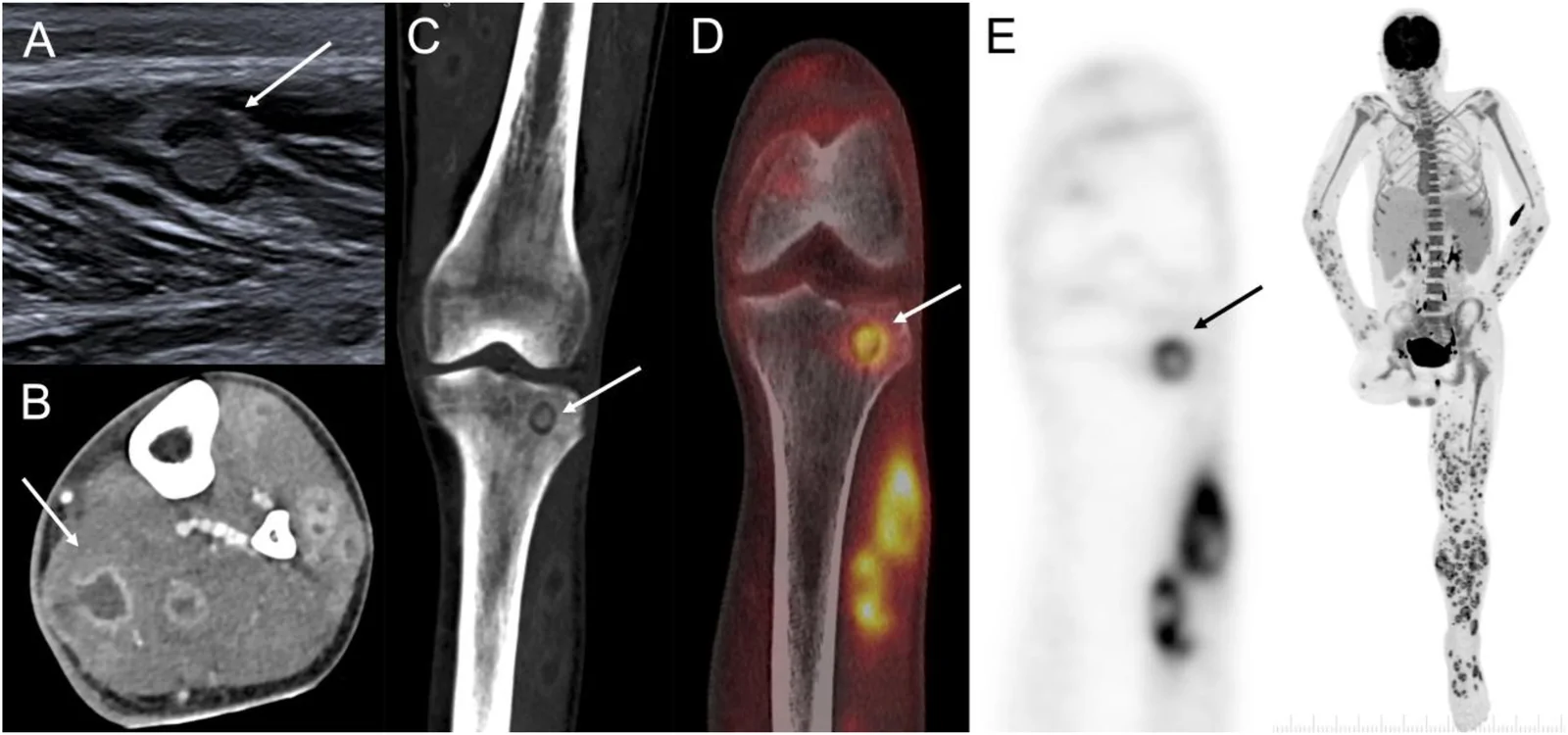
Follow‑up imaging demonstrating dynamic evolution during immune reconstitution: (A) Ultrasound showing well‑defined, rounded “target‑like” intramuscular lesions (white arrow). (B) Axial contrast‑enhanced CT showing interval progression of intramuscular fluid collections with peripheral rim enhancement (white arrow). (C) Coronal contrast‑enhanced CT of the left leg demonstrating a nodular lesion on tibial bone with central preserved density (sequestrum) and hypodense halo (white arrow). (D–E) Follow-up ^18^F‑FDG PET/CT demonstrating an increased number of soft‑tissue lesions with peripheral uptake compared to baseline (black and white arrows).

Over the following 2 weeks, multiple nodular bone lesions developed that were not present on the initial imaging studies. CT and PET/CT showed central hypometabolic sclerotic foci surrounded by hypermetabolic halos (Fig. 4C), and MRI demonstrated central fatty sequestra with surrounding inflammatory changes (Fig. 5). Repeat soft-tissue biopsies showed persistent inflammation but no fungal elements, and cultures—including calcofluor and molecular testing—were negative, findings suggestive of immune reconstitution inflammatory syndrome (IRIS). Surgical resection of bone sequestra was performed, antifungal therapy was continued, and the patient achieved sustained clinical recovery.

**Fig. 5:**
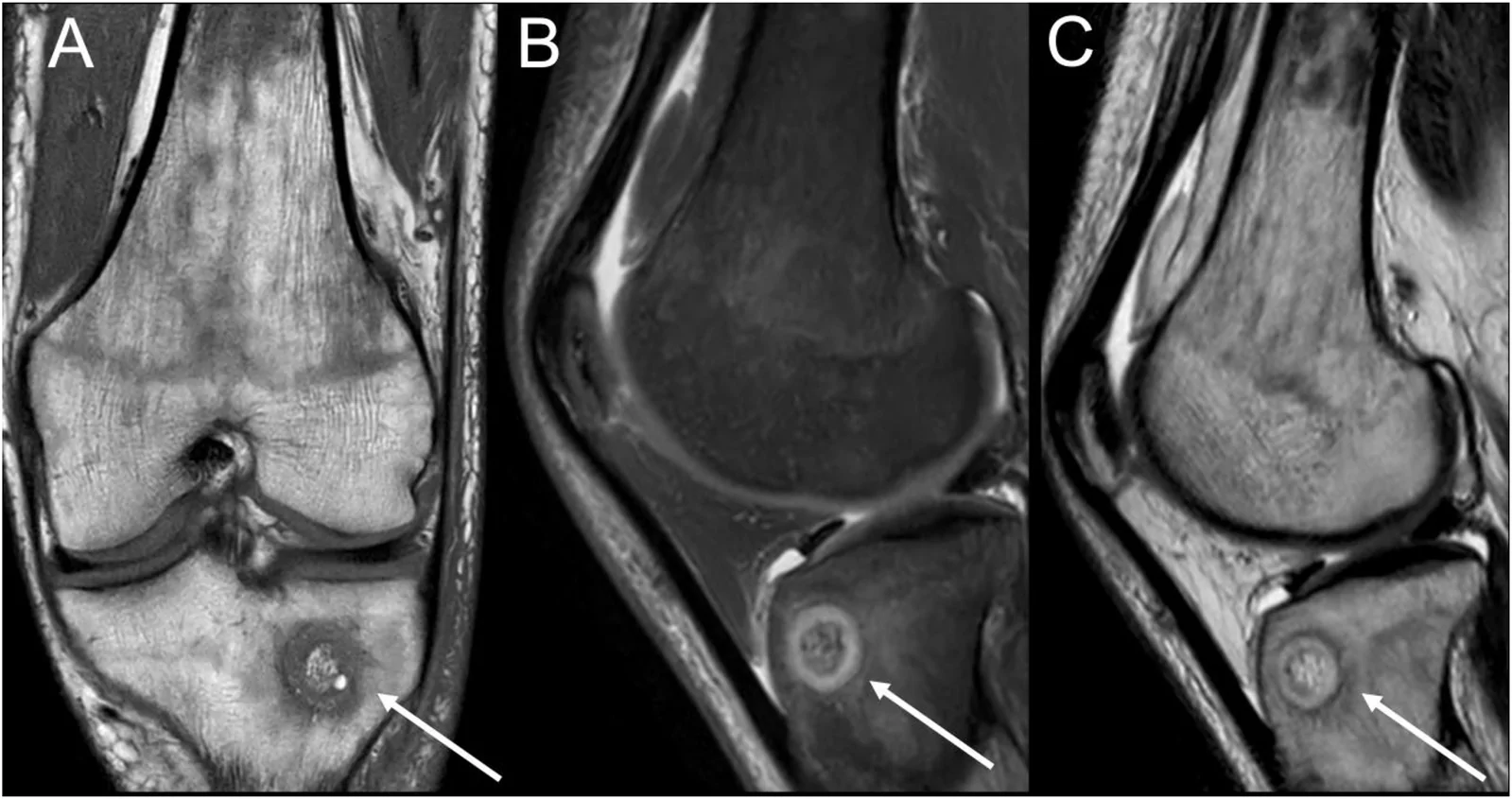
Left knee MRI (A, coronal T1-weighted; B, sagittal PD fat-suppressed; C, sagittal T2-weighted) demonstrating a well-defined, central bone sequestrum showing fat signal (white arrow) with a peripheral T1 hypointense rim, hyperintense on fluid-sensitive sequences (B, C), and associated marrow and adjacent soft tissue edema.

## Discussion

This case highlights the critical importance of early diagnosis in invasive fusariosis, a life-threatening condition with high mortality when diagnosis and treatment are delayed [4]. Multimodality imaging is central not only for detection but also for monitoring disease evolution. Familiarity with common sites of involvement, imaging characteristics, and dynamic lesion patterns is essential for accurate interpretation.

Contrast-enhanced CT and ^18^F-FDG PET/CT provide complementary information. CT offers detailed anatomical assessment, detects abscesses and necrotic tissue, and facilitates image-guided biopsy, while ^18^F-FDG PET/CT provides functional insight into metabolic activity related to host-pathogen interactions and treatment response [4]. Sequential ^18^F-FDG PET/CT, performed at baseline, after antifungal therapy initiation, and during follow-up, may reveal normalization of radiotracer uptake before morphological resolution [5,6]. However, distinguishing active infection from inflammatory changes can be challenging, as both may demonstrate increased uptake [7].

Ultrasound is a valuable first-line tool for evaluating superficial soft-tissue lesions and guiding biopsy without ionizing radiation, although its utility is limited in deep-seated lesions or assessing overall disease burden. MRI is indispensable for detailed evaluation of paranasal sinuses, deep soft tissues, and bones. Its superior soft-tissue contrast enables early detection of bone marrow edema, abscesses, and osteomyelitis, often preceding changes visible on CT or ultrasound [5,8].

Imaging findings in invasive fungal infections are dynamic and closely linked to immune status. During profound neutropenia, lesions may be few, subtle, or atypical. With neutrophil recovery, lesions can increase in number and show more pronounced inflammatory features, as observed in this case with the development of the “target sign.” Apparent progression on imaging must therefore be interpreted in the broader clinical context, as it may reflect immune reconstitution rather than true disease worsening.

Finally, histopathological analysis remains essential [1,9]. While microbiological cultures are critical, pathology often provides definitive evidence, through identification of fungal morphology and angioinvasion [2]. Pathological evaluation also helps differentiate active infection from post-infectious inflammatory changes, aiding distinction between true progression and immune-mediated pseudoprogression.

## Conclusion

Invasive fusariosis remains a diagnostic challenge in immunocompromised patients due to its variable and evolving clinical and imaging features. Multimodality imaging is essential for diagnosis and follow-up, but must always be interpreted in conjunction with histopathological and microbiological data to differentiate active infection from immune reconstitution–related inflammatory changes.

This case highlights the dynamic musculoskeletal manifestations of disseminated fusariosis, particularly the development of a characteristic imaging “target sign,” and underscores the importance of integrated clinicoradiological-pathological assessment.

## Patient consent

The patient gave their informed consent to the publication of this case report.
